# Sugar as an Anti-Aphrodisiac

**Published:** 1849-05

**Authors:** 


*Sugar as an anti-aphrodisiac.*—Dr. Provencal, former *chef de clinique* of the medical school of Montpellier thus speaks of this remedy. Sugar in large doses, in a hygienic point of view, is according to my experience, the most powerful medicament that can be administered as an anti-aphrodisiac. Camphor, by its prompt and instantaneous action has, up to the present time, occupied the first rank, and it is with great justice that it has been considered the antidote of cantharides, which is pre-eminently the aphrodisiac remedy.

Experience has proved to me that sugar in large doses is a truly powerful aliment and medicine, being greatly superior to camphor, since it unites the double property of paralyzing, as a medicine, the venereal ardor, and of repairing, as an aliment, its unpleasant consequences. The dose of sugar is one pound a day dissolved in a quart of water, milk or wine and taken at meals. In cases of masturbation, seminal losses, as well as in all cases of feebleness, wherever, in a word, it is necessary to repair the forces for which water and milk would be inadequate, I administer it in wine.

In cases of general irritability of the system, as is frequently observed among members of religious bodies, and in cases of priapism, I prescribe the sugar dissolved in cold water; finally in cases of excitation or irritation of the sexual organs complicated with irritation of the lungs, the sugar is given in milk or lukewarm ptisan of barley, if the milk prove difficult of digestion.

